# Dataset describing the genome wide effects on transcription resulting from alterations in the relative levels of the bZIP transcription factors Atf1 and Pcr1 in *Schizosaccharomyces pombe*

**DOI:** 10.1016/j.dib.2022.108034

**Published:** 2022-03-08

**Authors:** Sohini Basu, Priyanka Sarkar, Suchismita Datta, Geetanjali Sundaram

**Affiliations:** Department of Biochemistry, University of Calcutta, 35, Ballygunge Circular Road, Kolkata, WB 700019, India

**Keywords:** *S. pombe*, Atf1, Pcr1, Stress response, Transcriptome, bZIP

## Abstract

*Schizosaccharomyces pombe* has been used as an excellent model for studying eukaryotic cell cycle regulation and stress responses. The bZIP transcription factors Atf1(ATF2 homolog) and Pcr1(CREB homolog) have been shown to be important for regulating the expression of genes related to both stress response and cell cycle. Pcr1 has in fact been implicated as a determining factor in the segregation of the cell cycle and stress response related functions of Atf1. Interestingly Atf1 and Pcr1 levels are known to vary during the cell cycle thus giving rise to the possibility that their relative levels can influence the periodic transcriptional program of the cell. Here we report our observations on the changes in transcriptome of *S. pombe* cells which have been genetically manipulated to create relative differences in the levels of Atf1 and Pcr1. These results highlight new information regarding the potential role of Atf1 and Pcr1 in orchestrating the integration of the transcriptional programs of cell cycle and stress response.

**Specifications Table**

**Table:** 

Subject	Biology
Specific subject area	Molecular biology
Type of data	Table Venn diagram Graph
How the data were acquired	Data was acquired using Next Generation Sequencing TruSeq stranded mRNA preparation protocol was used to capture RNA, then the mRNA was purified and the cDNA library was prepared. The RNA sequence data were generated as a Fastq file. The quality of the data was checked. Read mapping to the reference genome was done using Cuffdiff. Gene ontology annotations were assigned using Uniprot, and the data analysis report was created.
Data format	Analyzed
Description of Data Collection	The *S. pombe* cells used in this study include *wild-type, Δatf1* and *Δatf1Δpcr1, wt* cells overexpressing Pcr1 and *Δatf1* cells overexpressing Pcr1*.* Total RNA was isolated from all these cells and processed for transcriptome sequencing.
Data Source Location	• Institution: University of Calcutta • City/Town/Region: Kolkata, West Bengal • Country: India
Data accessibility	Repository name: Gene Expression Omnibus (GEO) NCBI Sequence Read Archive (SRA) Data identification number: GSE175982 Direct URL to data: https://www-ncbi-nlm-nih-gov.brum.beds.ac.uk/geo/query/acc.cgi?acc=GSE175982

**Value of the Data**

• The data reflects the gene expression landscape of *S. pombe* strains with altered levels of Atf1 and Pcr1, which are homologs of mammalian ATF2 and CREB, thus expanding our knowledge about individual functional roles of these two transcription factors in a living cell. Deregulation of both ATF2 and CREB is associated with multiple developmental disorders and tumorigenesis. Clear understanding of the interplay between these two transcription factors and its effect on the cell's transcription program is therefore very important.
• The analysis of the data presented in this report identifies genes whose expression can be regulated by Pcr1 independently of Atf1. This is an important information as in earlier reports Pcr1 functions have been mostly characterized in the context of promoter specificity of Atf1.
• Analysis of this dataset clearly shows the control exerted by Pcr1 on the expression of genes important for many important fundamental biological processes like stress response and cell cycle.
• These data provide an entry point into investigations aimed at understanding how balance of the two transcription factors Atf1 and Pcr1 can regulate cell fate and proliferation. Extrapolation of these data can also facilitate studies aimed at understanding the contribution of ATF2 and CREB in disease progression.

## 1 Data Description

**Fig. 1:**
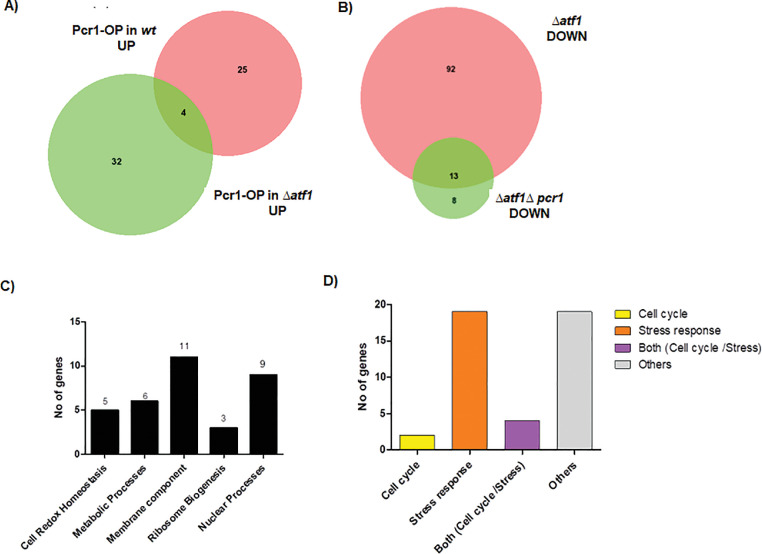
*Transcriptome analysis reveals targets which are differentially upregulated by Pcr1***.** Analysis was done using BioVenn [7] to find out the overlaps between different datasets. (A) Overlap between Pcr1-OP in *wt* and Pcr1-OP in *Δatf1* cells showed 36 genes to be upregulated by Pcr1, independent of regulation by Atf1. (B). Upon comparing *Δatf1* and *Δatf1Δpcr1*, we found 8 genes to be uniquely downregulated in the latter, which could be considered as targets induced solely by Pcr1. (C) Genes found to be positively upregulated by Pcr1 independently of Atf1 were sorted into significant functional clusters obtained from DAVID based analysis of genes represented in Table 9. (D) Graph represents the association of the genes positively upregulated by Pcr1 independently of Atf1 with cell cycle and/ or stress response or both.

**Fig. 2:**
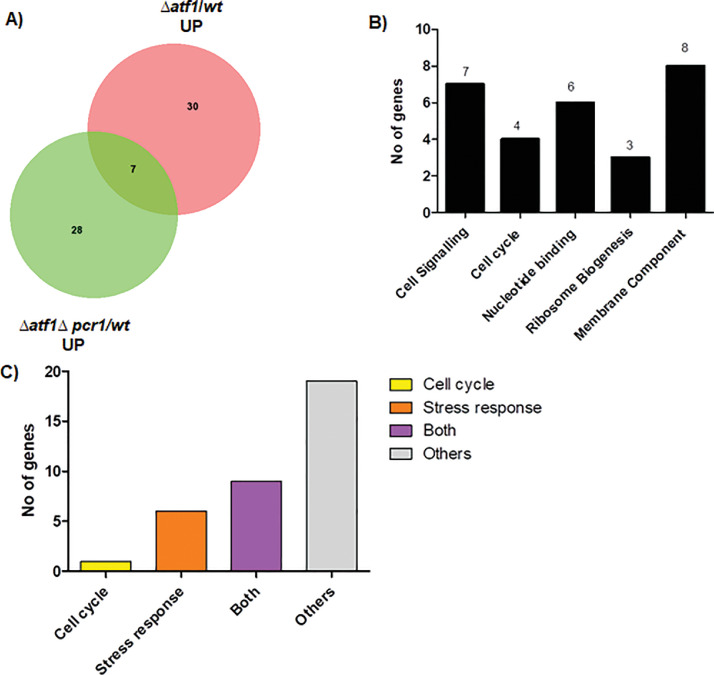
*Transcriptome analysis reveals targets which are differentially downregulated by Pcr1* (A) 28 genes that were found to be upregulated in *Δatf1Δpcr1* cells are possible targets negatively regulated by Pcr1. B) Categories with the highest number of genes in the significant functional clusters obtained from DAVID based analysis are represented for the 35 genes found to be downregulated by Pcr1. (C) Genes were sorted on the basis of their function in the cell cycle and /or stress response. Graph reflects the distribution of genes in each category.

**Fig. 3:**
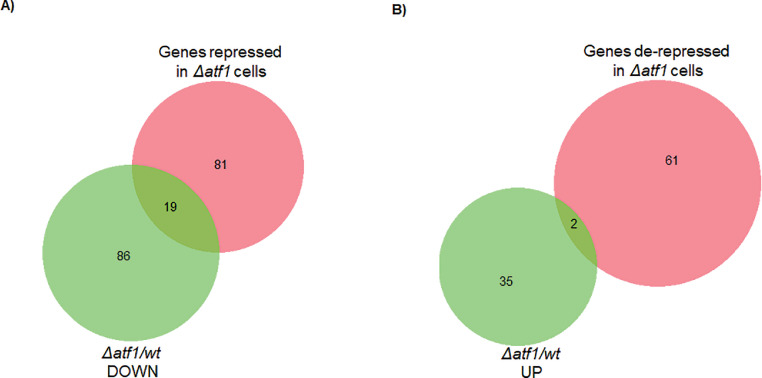
*Comparative analysis of transcriptome reveals new targets of Atf1***.** The genes found to be differentially regulated by Atf1 in our analysis have been compared with published data [3]. (A) Comparing genes downregulated in *∆atf1 S. pombe* cells of our analysis with genes already reported as targets of Atf1, we found 75 unique genes to be upregulated by Atf1, which can be identified as new targets of the transcription factor. (B) Comparison between genes upregulated in *∆atf1* cells and genes reported to be de-repressed in *∆atf1* cells have revealed 34 new targets of Atf1, which are uniquely downregulated in our analysis.

Studies done in our lab have established Pcr1 to be important in combating stress responses and to have contrasting outcomes on cell cycle progression [1]. In this study, we used genetic manipulations to vary the relative levels of Atf1 and Pcr1 in *S. pombe* cells. To study the effects of increase in Pcr1 levels, it was overexpressed in *wt* and Δ*atf1* cells and the transcriptional profiles of these cells were characterised. The effect of decrease in Atf1 levels was studied by comparing the gene expression profile of *wt* and *Δatf1* cells. The effect of complete absence of both these transcription factors was studied by comparing the transcriptomes of *wt* and *Δatf1Δpcr1* cells.The group of genes identified to be induced and repressed in each set of experiments are reported in (Table 1, Table 2, Table 3, Table 4, Table 5, Table 6, Table 7, Table 8). We performed a comparative analysis between the datasets obtained between different backgrounds, looking for unique genes . We found only 4 genes to be commonly upregulated by Pcr1 overexpression in both *wt* and *∆atf1* cells (Fig. 1A). 8 genes were found to be downregulated only in the double mutant (Fig. 1B). Comparison of these data revealed the identity of genes that can be positively regulated by Pcr1 independently of Atf1 (Table 9). The genes found to be regulated independently by Pcr1 were then analyzed to identifiy the cellular processes associated with the gene expression changes using DAVID [2,3]. DAVID analysis classified the genes to be important in several biological processes(Fig. 1C). The known expression changes of these genes during stress response [4] and cell division [5] was then looked up and the genes were then classified into Stress reponse and Cell cycle categories. We found that groups of genes are important during the stress response, the cell cycle, or both (Fig. 1D). 28 genes were found to be upregulated only in the *∆atf1∆pcr1* when compared to genes upregulated in *∆atf1* cells (Fig. 2A). DAVID analysis identified several pathways that are downregulated by Pcr1 (Fig. 2B). These genes were also classified according to their previously known association with cell cycle and stress response (Fig. 2C). Genes that are downregulated by Pcr1 independently of Atf1 are listed in Table 10. We compared the genes regulated by Pcr1 (Tables 9, 10) with those of the existing datasets of Atf1 dependent gene expression from studies previously conducted by us and other groups [4,6]. This comparison reveals that there are a few genes whose expression is regulated in a contrasting manner by Atf1 and Pcr1 (Tables 11, 12). We compared our gene list obtained from this study with existing data for Atf1-dependent gene expression [4] and found 75 new genes that are upregulated by Atf1 and 34 new genes that are downregulated by it (Fig. 3A, B) in absence of stress.

**Table 1:** List of genes upregulated during Pcr1 overexpression in *wt S. pombe* cells.

Gene ID	Gene Symbol	Gene Function
SPAC21E11.03c	*pcr1*	DNA-binding transcription factor Pcr1
SPAC19G12.16c	*adg2*	conserved fungal cell surface protein, Kre9/Knh1 family
SPAC212.11	*tlh1*	RecQ type DNA helicase
SPBC1105.05	*exg1*	cell wall glucan 1,6-beta-glucosidase Exg1
SPAPB1E7.04c	SPAPB1E7.04c	chitinase
SPSNORNA.32	*sno12*	box H/ACA small nucleolar RNA 12/snR99
SPBC1348.14c	*ght7*	plasma membrane hexose transmembrane transporter Ght7
SPNCRNA.942	SPNCRNA.942	intergenic RNA (predicted)
SPRRNA.02	*rns*	small subunit (15S) rRNA, rns
SPAC186.09	*pdc102*	pyruvate decarboxylase
SPBPB2B2.08	SPBPB2B2.08	conserved fungal protein
SPNCRNA.532	SPNCRNA.532	non-coding RNA (predicted)
SPAC1F8.05	*isp3*	spore wall structural constituent Isp3
SPAC1039.11c	*gto1*	alpha-glucosidase
SPAC23A1.02c	*ted1*	GPI-remodeling mannose-ethanolamine phosphate phosphodiesterase Ted1
SPCPB1C11.01	*amt1*	plasma membrane ammonium transmembrane transporter
SPAC20G8.05c	*cdc15*	F-BAR domain protein Cdc15
SPCC306.11	SPCC306.11	Schizosaccharomyces specific protein, uncharacterized
SPAC13G7.04c	*mac1*	plasma membrane anchored protein, claudin family, predicted membrane sensor Mac1
SPRRNA.46	SPRRNA.46	18S ribosomal RNA
SPNCRNA.1374	*cta3-antisense-1*	antisense RNA (predicted)
SPAPB1E7.05	*gde1*	glycerophosphoryl diester phosphodiesterase Gde
SPBC11C11.05	*SPBC11C11.05*	conserved fungal cell wall protein, Kre9/Knh1 family
SPCC1235.13	*ght6*	plasma membrane glucose/fructose:proton symporter Ght6
SPBC14C8.01c	*cut2*	sister chromatid separation inhibitor, securin
SPAC821.09	*eng1*	cell septum surface endo-1,3-beta-glucanase Eng1
SPAC1006.08	*etd1*	Spg1-binding protein Etd1
SPBP26C9.03c	*fet4*	plasma membrane iron/zinc ion transmembrane transporter
SPBC1685.14c	*vid27*	WD repeat protein, Vid27 family, conserved in fungi and plants

**Table 2:** List of genes downregulated during Pcr1 over expression in *wt S. pombe* cells.

Gene ID	Gene Symbol	Gene Function
SPAP8A3.10	*ups1*	mitochondrial phosphatidic acid transfer protein Ups1
SPAP27G11.13c	*nop10*	box H/ACA snoRNP complex protein

**Table 3:** List of genes upregulated in *Δatf1* cells.

Gene ID	Gene Symbol	Gene Function
SPAC1F8.04c	SPAC1F8.04c	hydrolase, implicated in cellular detoxification
SPRRNA.01	*rnl*	large subunit (21S) rRNA, rnl
SPRRNA.02	*rns*	small subunit (15S) rRNA, rns
SPMIT.06	SPMIT.06	mitochondrial DNA binding endonuclease (intron encoded)
SPCC576.01c	*xan1*	alpha-ketoglutarate-dependent xanthine dioxygenase Xan1
SPCC4B3.10c	*ipk1*	inositol 1,3,4,5,6-pentakisphosphate (IP5) kinase
SPCC1223.09	*uro1*	uricase Uro1
SPAPB1E7.04c	SPAPB1E7.04c	chitinase
SPAC1039.02	SPAC1039.02	extracellular 5′-nucleotidase, human NT5E family
SPAC1399.01c	SPAC1399.01c	nucleobase transmembrane transporter
SPAC19G12.16c	*adg2*	conserved fungal cell surface protein, Kre9/Knh1 family
SPAC20G8.09c	*nat10*	rRNA/tRNA cytidine N-acetyltransferase
SPCPB1C11.01	*amt1*	plasma membrane ammonium transmembrane transporter
SPAC1039.01	SPAC1039.01	amino acid transmembrane transporter
SPCC757.13	SPCC757.13	dipeptide transmembrane transporter
SPAC25B8.13c	*isp7*	2-OG-Fe(II) oxygenase superfamily protein
SPBC31E1.06	*bms1*	GTP binding protein Bms1
SPBC56F2.04	*utp20*	U3 snoRNP protein Utp20
SPAC1002.17c	*urg2*	uracil phosphoribosyltransferase
SPBC4C3.05c	*nuc1*	DNA-directed RNA polymerase I complex large subunit Nuc1
SPAC20G8.06	*not1*	CCR4-Not complex scaffold subunit 1
SPBC2G2.08	*ade9*	C-1-tetrahydrofolatesynthase/methylenetetrahydrof olatedehydrogenase/methylenetetrahydrofolatecyclohydrolase/ formyltetrahydrofolatesynthetase Ade9
SPAC869.05c	*sul2*	plasma membrane sulfate transmembrane transporter Sul2
SPBC12C2.10c	*pst1*	Clr6 histone deacetylase complex subunit Pst1
SPBC27B12.11c	*pho7*	DNA-binding transcription factor Pho7
SPBC342.03	*gas4*	spore wall 1,3-beta-glucanosyltransferase Gas4
SPBC29B5.02c	*isp4*	plasma membrane OPT oligopeptide transmembrane transporter family Isp4
SPAC19D5.04	*ptr1*	HECT-type ubiquitin-protein ligase E3 Ptr1
SPAC3G6.01	*hrp3*	CHD family chromatin remodeller Hrp3
SPAC19B12.01	SPAC19B12.01	TPR repeat protein, human TTC27 ortholog
SPAC4F10.09c	*noc1*	ribosome biogenesis protein Noc1
SPCC1183.07	*rrp5*	U3 snoRNP-associated protein Rrp5
SPBC11C11.02	*imp2*	F-BAR domain protein Imp2
SPBC800.10c	*ede1*	EPS15 repeat family actin cortical patch component Ede1
SPAC821.09	*eng1*	cell septum surface endo-1,3-beta-glucanase Eng1
SPAC4H3.11c	*ppc89*	spindle pole body protein Ppc89
SPBC1826.01c	*mot1*	TATA-binding protein-associated transcription initiation factor Mot1

**Table 4:** List of genes downregulated in *Δatf1* cells.

Gene ID	Gene Symbol	Gene Function
SPAP8A3.04c	*hsp9*	heat shock protein Hsp9
SPAC19A8.16	*prl65*	tudor domain superfamily protein
SPBC32F12.03c	*gpx1*	H2O2 scavenger glutathione peroxidase Gpx1
SPCC1393.12	SPCC1393.12	Schizosaccharomyces specific protein, uncharacterized
SPAC22H10.13	*zym1*	metallothionein Zym1
SPAC977.16c	*dak2*	dihydroxyacetone kinase Dak2
SPNCRNA.103	*sme2*	meiosis-specific Smp foci lncRNA Sme2, long isoform
SPAC343.12	*rds1*	ferritin related conserved fungal protein
SPCC757.03c	*hsp3101*	glyoxylase III Hsp3101
SPAC4H3.08	SPAC4H3.08	3-hydroxyacyl-CoA dehydrogenase
SPBC359.06	*mug14*	adducin, involved in actin cytoskeleton organization
SPNCRNA.570	SPNCRNA.570	non-coding RNA (predicted)
SPAPB1A11.02	SPAPB1A11.02	esterase/lipase
SPBPB2B2.06c	SPBPB2B2.06c	extracellular 5′-nucleotidase, human NT5E family
SPAC15E1.02c	SPAC15E1.02c	DUF1761 family protein, conserved unknown
SPAC1F8.01	*ght3*	plasma membrane gluconate:proton symporter Ght3
SPAC19G12.09	SPAC19G12.09	NADH/NADPH-dependent indole-3-acetaldehyde reductase, implicated in cellular detoxification
SPAC4F10.20	*grx1*	glutaredoxin Grx1
SPBC56F2.15	*tam13*	Schizosaccharomyces specific protein, uncharacterized
SPBC16E9.16c	*lsd90*	Lsd90 protein
SPBC21C3.19	*rtc3*	SBDS family protein Rtc3
SPBC215.05	*gpd1*	glycerol-3-phosphate dehydrogenase Gpd1
SPBC1289.14	SPBC1289.14	adducin
SPAC3G6.07	SPAC3G6.07	Schizosaccharomyces specific protein, uncharacterized
SPAPB24D3.10c	*agl1*	maltose alpha-glucosidase Agl1
SPBPB21E7.08	SPBPB21E7.08	pseudogene
SPNCRNA.1255	SPNCRNA.1255	intergenic RNA (predicted)
SPAC26F1.07	SPAC26F1.07	NADPH-dependent aldo-keto reductase
SPBC725.10	*tps0*	mitochondrial outer membrane protein, TspO/MBR-related, implicated in lipid/sterol transport, tspO
SPCPB16A4.07	*smp4*	Stm1/Oga1 family protein Smp4
SPNCRNA.1223	*SPCC191.10-antisense-1*	antisense RNA (predicted)
SPCPB16A4.06c	SPCPB16A4.06c	Schizosaccharomyces specific protein, uncharacterized
SPCC338.12	*pbi2*	vaculoar proteinase B inhibitor Pbi2
SPBC1198.14c	*fbp1*	fructose-1,6-bisphosphatase Fbp1
SPBC11C11.06c	SPBC11C11.06c	Schizosaccharomyces specific protein, uncharacterized
SPAC23C4.11	*atp18*	F1-FO ATP synthase subunit J
SPBC713.11c	*pmp3*	plasma membrane proteolipid Pmp3
SPAC29B12.13	SPAC29B12.13	CENP-V, S-(hydroxymethyl)glutathione synthase
SPCC330.06c	*pmp20*	thioredoxin-related chaperone Pmp20
SPAC11D3.01c	SPAC11D3.01c	Con-6 family conserved fungal protein
SPBC16A3.02c	SPBC16A3.02c	mitochondrial CH-OH group oxidoreductase, human RTN4IP1 ortholog, implicated in mitochondrial organization or tethering
SPAC977.15	SPAC977.15	dienelactone hydrolase family, implicated in cellular detoxification
SPCC757.07c	*ctt1*	catalase
SPNCRNA.445	SPNCRNA.445	non-coding RNA
SPAC10F6.06	*vip1*	RNA-binding protein Vip1
SPAC25G10.06	*rps2801*	40S ribosomal protein S28
SPCC794.01c	*gcd1*	glucose dehydrogenase Gcd1
SPBC26H8.14c	*cox17*	mitochondrial copper chaperone for cytochrome c oxidase Cox17
SPBC3E7.02c	*hsp16*	heat shock protein Hsp16
SPBC215.11c	SPBC215.11c	aldo/keto reductase, unknown biological role
SPBC17D1.17	*tam11*	Schizosaccharomyces specific protein, uncharacterized
SPNCRNA.1436	SPNCRNA.1436	non-coding RNA
SPBC725.03	SPBC725.03	pyridoxamine 5′-phosphate oxidase
SPAC3G9.11c	*pdc201*	pyruvate decarboxylase
SPBC32H8.07	*git5*	heterotrimeric G protein beta (WD repeat) subunit Git5
SPAC9E9.04	SPAC9E9.04	bcap family homolog, implicated in vesicle-mediated transport
SPAC15A10.05c	*mug182*	NADHX epimerase
SPAC4G9.12	*idn1*	gluconokinase
SPBC23G7.16	*ctr6*	vacuolar copper exporter Ctr6
SPBC21B10.04c	*nrf1*	vacuolar transporter chaperone (VTC) complex, GTPase regulator subunit Nrf1
SPCC965.06	*osr2*	potassium channel, beta subunit, aldo-keto reductase
SPNCRNA.906	*snR30*	non-coding RNA
SPAC823.17	*tom6*	mitochondrial TOM complex subunit Tom6
SPAC688.16	SPAC688.16	human TMEM254 ortholog
SPAC186.05c	*gdt1*	Golgi calcium and manganese antiporter Gdt1
SPBC660.05	*wwm3*	WW domain containing conserved fungal protein Wwm3
SPBC2A9.02	SPBC2A9.02	NADH-dependent glycolaldehyde/furfural/butyraldehyde/propylaldehydealdehyde reductase
SPAC22F8.05	SPAC22F8.05	alpha,alpha-trehalose-phosphate synthase
SPAC4G8.02c	*sss1*	translocon gamma subunit Sss1
SPCC794.04c	SPCC794.04c	amino acid transmembrane transporter
SPAC26F1.14c	*aif1*	mitochondrial inner membrane anchored oxidoreductase
SPAC4F8.10c	*stg1*	SM22/transgelin-like actin modulating protein Stg1
SPBC30D10.14	SPBC30D10.14	dienelactone hydrolase family
SPAC27D7.09c	SPAC27D7.09c	But2 family protein, similar to cell surface molecules
SPBC337.08c	*ubi4*	protein modifier, ubiquitin
SPAC1705.02	SPAC1705.02	SERF family protein, DUF, human 4F5S homolog, implicated in mRNA splicing
SPBP4H10.12	SPBP4H10.12	protein with a role in ER insertion of tail-anchored membrane proteins
SPAC1782.07	*qcr8*	ubiquinol-cytochrome-c reductase complex subunit 7
SPBC23G7.10c	SPBC23G7.10c	NADH-dependent flavin oxidoreductase, implicated in cellular detoxification from family members
SPAC3G6.13c	*rpl4101*	60S ribosomal protein L41
SPBC800.14c	SPBC800.14c	mitochondrial DUF1772 family protein, multimembrane spanning anthrone oxygenase-like
SPCC191.01	SPCC191.01	Schizosaccharomyces specific protein, uncharacterized
SPBC4B4.05	*smg1*	Sm snRNP core protein Smg1
SPAC922.04	SPAC922.04	Schizosaccharomyces specific protein, uncharacterized
SPAPJ691.03	*mic10*	MICOS complex subunit Mic10
SPBC3B9.13c	*rpp102*	ribosomal protein P1 Rpp102
SPBC405.04c	*ypt7*	GTPase Ypt7
SPAC2F3.05c	SPAC2F3.05c	xylose and arabinose reductase
SPNCRNA.844	SPNCRNA.844	intergenic RNA (predicted)
SPAC4D7.02c	*pgc1*	phosphatidylglycerol phospholipase C Pgc1
SPAC11D3.19	SPAC11D3.19	*Schizosaccharomyces pombe* specific protein
SPCC16A11.15c	SPCC16A11.15c	Schizosaccharomyces specific protein, uncharacterized
SPAC1F8.08	SPAC1F8.08	*Schizosaccharomyces pombe* specific protein, uncharacterized
SPAC1F12.10c	SPAC1F12.10c	NADPH-hemoprotein reductase
SPAP27G11.13c	*nop10*	box H/ACA snoRNP complex protein
SPAC4H3.03c	SPAC4H3.03c	glucan 1,4-alpha-glucosidase
SPAC6G9.07c	*arc4*	ARP2/3 actin-organizing complex subunit Arc4
SPAC23H3.02c	*ini1*	RING finger-like protein Ini1
SPAC19B12.06c	*rbd4*	rhomboid family protease, unknown biological role, associated with COP1 coated vesicle
SPAC26F1.10c	*pyp1*	protein tyrosine phosphatase Pyp1
SPAC630.11	*vps55*	vacuolar sorting protein Vps55
SPCC24B10.05	*tim9*	Tim9-Tim10 complex subunit Tim9
SPAPB24D3.08c	SPAPB24D3.08c	NADP-dependent oxidoreductase, implicated in cellular detoxification
SPAC6F12.04	*tvp15*	COPI-coated vesicle associated protein
SPCC663.02	*wtf14*	wtf element Wtf14

**Table 5:** List of genes upregulated during Pcr1 overexpression in *Δatf1* background.

Gene ID	Gene Symbol	Gene Function
SPBC32F12.03c	*gpx1*	H2O2 scavenger glutathione peroxidase Gpx1
SPBPB2B2.06c	SPBPB2B2.06c	extracellular 5′-nucleotidase, human NT5E family
SPAC19A8.16	*prl65*	tudor domain superfamily protein
SPBC23G7.15c	*rpp202*	60S acidic ribosomal protein P2
SPNCRNA.103	*sme2*	meiosis-specific Smp foci lncRNA Sme2, long isoform
SPAC4F10.20	*grx1*	glutaredoxin Grx1
SPAC22H10.13	*zym1*	metallothionein Zym1
SPAC1F8.01	*ght3*	plasma membrane gluconate:proton symporter Ght3
SPAC21E11.03c	*pcr1*	DNA-binding transcription factor Pcr1
SPBC56F2.15	*tam13*	Schizosaccharomyces specific protein Tam13
SPCC1393.12	SPCC1393.12	Schizosaccharomyces specific protein, uncharacterized
SPBC11C11.06c	SPBC11C11.06c	Schizosaccharomyces specific protein, uncharacterized
SPNCRNA.570	SPNCRNA.570	non-coding RNA (predicted)
SPCPB16A4.07	SPCPB16A4.07	Stm1/Oga1 family protein Smp4
SPBPB21E7.08	SPBPB21E7.08	pseudogene
SPAC15E1.02c	SPAC15E1.02c	DUF1761 family protein
SPNCRNA.1436	SPNCRNA.1436	non-coding RNA
SPCC338.12	*pbi2*	vaculoar proteinase B inhibitor Pbi2
SPAC19G12.09	*SPAC19G12.09*	NADH/NADPH-dependent indole-3-acetaldehyde reductase, implicated in cellular detoxification
SPNCRNA.942	*SPNCRNA.942*	intergenic RNA (predicted)
SPBC359.06	*mug14*	adducin, involved in actin cytoskeleton organization
SPNCRNA.98	*srp7*	7SL signal recognition particle component
SPSNORNA.32	*sno12*	box H/ACA small nucleolar RNA 12/snR99
SPBC725.10	*tps0*	mitochondrial outer membrane protein, TspO/MBR-related, implicated in lipid/sterol transport, tspO
SPAC26F1.07	SPAC26F1.07	NADPH-dependent aldo-keto reductase
SPAC23C4.11	atp18	F1-FO ATP synthase subunit J
SPAC9E9.04	SPAC9E9.04	bcap family homolog, implicated in vesicle-mediated transport
SPNCRNA.808	SPNCRNA.808	intergenic RNA (predicted)
SPBC26H8.14c	cox17	mitochondrial copper chaperone for cytochrome c oxidase Cox17
SPAC1F8.03c	*str3*	plasma membrane heme transmembrane transporter Str3
SPAC1F8.05	*isp3*	spore wall structural constituent Isp3
SPBC21B10.04c	nrf1	vacuolar transporter chaperone (VTC) complex, GTPase regulator subunit Nrf1
SPBC215.11c	SPBC215.11c	aldo/keto reductase, unknown biological role
SPBC11B10.10c	pht1	histone H2A variant H2A.Z Pht1
SPAC4F8.10c	*stg1*	SM22/transgelin-like actin modulating protein Stg1
SPAC22F8.05	SPAC22F8.05	alpha,alpha-trehalose-phosphate synthase

**Table 6:** List of genes downregulated during Pcr1 overexpression in *Δatf1* background.

Gene ID	Gene Symbol	Gene Function
SPCC576.01c	*xan1*	alpha-ketoglutarate-dependent xanthine dioxygenase Xan1
SPCC1223.09	*uro1*	uricase Uro1
SPAC1002.19	*urg1*	GTP cyclohydrolase II Urg1
SPAC1039.02	SPAC1039.02	extracellular 5′-nucleotidase, human NT5E family
SPCC4B3.10c	*ipk1*	inositol 1,3,4,5,6-pentakisphosphate (IP5) kinase
SPAC56F8.03	SPAC56F8.03	translation initiation factor eIF5B Tif52
SPAC29B12.14c	SPAC29B12.14c	plasma membrane purine transmembrane transporter

**Table 7:** List of genes upregulated in *Δatf1Δpcr1* cells.

Gene ID	Gene Symbol	Gene Function
SPAC212.11	*tlh1*	RecQ type DNA helicase
SPAC19G12.16c	*adg2*	conserved fungal cell surface protein, Kre9/Knh1 family, Adg2
SPBC1348.14c	*ght7*	plasma membrane hexose transmembrane transporter Ght7
SPAPB1E7.04c	SPAPB1E7.04c	chitinase
SPBC1105.05	*exg1*	cell wall glucan 1,6-beta-glucosidase Exg1
SPRRNA.02	*15S_rRNA*	small subunit (15S) rRNA, rns
SPAC1039.11c	*gto1*	alpha-glucosidase
SPSNORNA.32	*sno12*	box H/ACA small nucleolar RNA 12/snR99
SPAC186.09	*pdc102*	pyruvate decarboxylase
SPAC19B12.02c	*gas1*	cell wall 1,3-beta-glucanosyltransferase Gas1
SPBC4F6.12	*pxl1*	paxillin-like protein Pxl1
SPRRNA.45	SPRRNA.45	18S ribosomal RNA
SPAC1F8.05	*isp3*	spore wall structural constituent Isp3
SPAC750.01	SPAC750.01	NADP-dependent aldo/keto reductase, unknown biological role, implicated in cellular detoxification
SPRRNA.46	SPRRNA.46	18S ribosomal RNA
SPMIT.06	SPMIT.06	mitochondrial DNA binding endonuclease (intron encoded)
SPNCRNA.532	SPNCRNA.532	non-coding RNA (predicted)
SPRRNA.44	SPRRNA.44	18S ribosomal RNA
SPAC20G8.05c	*cdc15*	F-BAR domain protein Cdc15
SPRRNA.01	*rnl*	large subunit (21S) rRNA, rnl
SPBPB2B2.13	*gal1*	galactokinase Gal1
SPNCRNA.942	SPNCRNA.942	intergenic RNA (predicted)
SPAPB1E7.05	*gde1*	glycerophosphoryl diester phosphodiesterase Gde1
SPAC13G7.04c	*mac1*	plasma membrane anchored protein, claudin family, predicted membrane sensor Mac1
SPCC306.11	SPCC306.11	Schizosaccharomyces specific protein, uncharacterized
SPNCRNA.1374	*cta3-antisense-1*	antisense RNA (predicted)
SPBC11C11.05	SPBC11C11.05	conserved fungal cell wall protein, Kre9/Knh1 family
SPAC23A1.02c	SPAC23A1.02c	GPI-remodeling mannose-ethanolamine phosphate phosphodiesterase Ted1
SPAC1006.08	*etd1*	Spg1-binding protein Etd1
SPBC1289.01c	*chr4*	SEL1/TPR repeat protein1, 3-beta-glucan synthase regulatory factor Chf3/Chr4
SPAC1F7.05	*cdc22*	ribonucleoside reductase large subunit Cdc22
SPBC1685.14c	*vid27*	WD repeat protein, Vid27 family, conserved in fungi and plants
SPAC821.09	*eng1*	cell septum surface endo-1,3-beta-glucanase Eng1
SPBC1289.04c	*pob1*	Boi family protein
SPBC31E1.06	*bms1*	GTP binding protein Bms1

**Table 8:** List of genes downregulated in *Δatf1Δpcr1* cells.

Gene ID	Gene Symbol	Gene Function
SPAC688.16	SPAC688.16	human TMEM254 ortholog
SPNCRNA.1255	SPNCRNA.1255	intergenic RNA (predicted)
SPAC29A4.12c	SPAC29A4.12c	Schizosaccharomyces specific protein, uncharacterized
SPBC660.05	*wwm3*	WW domain containing conserved fungal protein Wwm3
SPAPB18E9.05c	SPAPB18E9.05c	dubious
SPNCRNA.1223	*SPCC191.10-antisense-1*	antisense RNA (predicted)
SPBPB21E7.11	SPBPB21E7.11	*Schizosaccharomyces pombe* specific protein, uncharacterized
SPAP27G11.13c	*nop10*	box H/ACA snoRNP complex protein
SPAC19A8.16	*prl65*	tudor domain superfamily protein
SPAC513.03	*mfm2*	M-factor precursor Mfm2
SPBC56F2.15	*tam13*	Schizosaccharomyces specific protein, uncharacterized
SPCPB16A4.07	SPCPB16A4.07	Stm1/Oga1 family protein Smp4
SPAC15E1.02c	SPAC15E1.02c	DUF1761 family protein
SPBC26H8.14c	*cox17*	mitochondrial copper chaperone for cytochrome c oxidase Cox17
SPCC16C4.13c	*rpl1201*	60S ribosomal protein L12.1/L12A
SPAC823.17	*tom6*	mitochondrial TOM complex subunit Tom6
SPCC663.02	*wtf14*	wtf element Wtf14
SPBC1604.11	*atp17*	F1-FO ATP synthase subunit F
SPBC4B4.05	*smg1*	Sm snRNP core protein Smg1
SPCC31H12.04c	*rpl1202*	60S ribosomal protein L12.1/L12A
SPCC1259.05c	*cox9*	cytochrome c oxidase subunit VIIa

**Table 9:** List of genes upregulated by Pcr1 independent of Atf1.

Gene ID	Gene Symbol	Gene Function
SPBC32F12.03c	*gpx1*	H2O2 scavenger glutathione peroxidase Gpx1
SPBPB2B2.06c	SPBPB2B2.06c	extracellular 5′-nucleotidase, human NT5E family
SPAC19A8.16	*prl65*	tudor domain superfamily protein
SPBC23G7.15c	*rpp202*	60S acidic ribosomal protein P2
SPNCRNA.103	*sme2*	meiosis-specific Smp foci lncRNA Sme2, long isoform
SPAC4F10.20	*grx1*	glutaredoxin Grx1
SPAC22H10.13	*zym1*	metallothionein Zym1
SPAC1F8.01	*ght3*	plasma membrane gluconate:proton symporter Ght3
SPAC21E11.03c	*pcr1*	DNA-binding transcription factor Pcr1
SPBC56F2.15	*tam13*	Schizosaccharomyces specific protein, uncharacterized
SPCC1393.12	SPCC1393.12	Schizosaccharomyces specific protein, uncharacterized
SPBC11C11.06c	SPBC11C11.06c	Schizosaccharomyces specific protein, uncharacterized
SPNCRNA.570	SPNCRNA.570	non-coding RNA (predicted)
SPCPB16A4.07	*smp4*	Stm1/Oga1 family protein Smp4
SPBPB21E7.08	SPBPB21E7.08	pseudogene
SPAC15E1.02c	SPAC15E1.02c	DUF1761 family protein
SPNCRNA.1436	SPNCRNA.1436	non-coding RNA
SPCC338.12	*pbi2*	vaculoar proteinase B inhibitor Pbi2
SPAC19G12.09	SPAC19G12.09	NADH/NADPH-dependent indole-3-acetaldehyde reductase, implicated in cellular detoxification
SPNCRNA.942	SPNCRNA.942	intergenic RNA (predicted)
SPBC359.06	*mug14*	adducin, involved in actin cytoskeleton organization
SPNCRNA.98	*srp7*	7SL signal recognition particle component
SPSNORNA.32	*sno12*	box H/ACA small nucleolar RNA 12/snR99
SPBC725.10	*tps0*	mitochondrial outer membrane protein, TspO/MBR-related, implicated in lipid/sterol transport, tspO
SPAC26F1.07	SPAC26F1.07	NADPH-dependent aldo-keto reductase
SPAC23C4.11	*atp18*	F1-FO ATP synthase subunit J
SPAC9E9.04	SPAC9E9.04	bcap family homolog, implicated in vesicle-mediated transport
SPNCRNA.808	SPNCRNA.808	intergenic RNA (predicted)
SPBC26H8.14c	*cox17*	mitochondrial copper chaperone for cytochrome c oxidase Cox17
SPAC1F8.03c	*str3*	plasma membrane heme transmembrane transporter Str3
SPAC1F8.05	*isp3*	spore wall structural constituent Isp3
SPBC21B10.04c	*nrf1*	vacuolar transporter chaperone (VTC) complex, GTPase regulator subunit Nrf1
SPBC215.11c	SPBC215.11c	aldo/keto reductase, unknown biological role
SPBC11B10.10c	*pht1*	histone H2A variant H2A.Z Pht1
SPAC4F8.10c	*stg1*	SM22/transgelin-like actin modulating protein Stg1
SPAC22F8.05	SPAC22F8.05	alpha,alpha-trehalose-phosphate synthase
SPAPB18E9.05c	SPAPB18E9.05c	dubious
SPBPB21E7.11	SPBPB21E7.11	*Schizosaccharomyces pombe* specific protein, uncharacterized
SPAC513.03	*mfm2*	M-factor precursor Mfm2
SPCC16C4.13c	*rpl1201*	60S ribosomal protein L12.1/L12A
SPBC1604.11	*atp17*	F1-FO ATP synthase subunit F
SPCC31H12.04c	*rpl1202*	60S ribosomal protein L12.1/L12A
SPCC1259.05c	*cox9*	cytochrome c oxidase subunit VIIa
SPAC29A4.12	*mug108*	Schizosaccharomyces specific protein, uncharacterized

**Table 10:** List of genes repressed by Pcr1 independent of Atf1.

Gene ID	Gene Symbol	Gene Function
SPAC212.11	*tlh1*	RecQ type DNA helicase
SPBC1348.14c	*ght7*	plasma membrane hexose transmembrane transporter Ght7
SPBC1105.05	*exg1*	cell wall glucan 1,6-beta-glucosidase Exg1
SPAC1039.11c	*gto1*	alpha-glucosidase
SPSNORNA.32	*sno12*	box H/ACA small nucleolar RNA 12/snR99
SPAC186.09	*pdc102*	pyruvate decarboxylase
SPAC19B12.02c	*gas1*	cell wall 1,3-beta-glucanosyltransferase Gas1
SPBC4F6.12	*pxl1*	paxillin-like protein Pxl1
SPRRNA.45	SPRRNA.45	18S ribosomal RNA
SPAC1F8.05	*isp3*	spore wall structural constituent Isp3
SPAC750.01	SPAC750.01	NADP-dependent aldo/keto reductase, unknown biological role, implicated in cellular detoxification
SPRRNA.46	SPRRNA.46	18S ribosomal RNA
SPNCRNA.532	SPNCRNA.532	non-coding RNA (predicted)
SPRRNA.44	SPRRNA.44	18S ribosomal RNA
SPAC20G8.05c	*cdc15*	F-BAR domain protein Cdc15
SPBPB2B2.13	*gal1*	galactokinase Gal1
SPNCRNA.942	SPNCRNA.942	intergenic RNA (predicted)
SPAPB1E7.05	*gde1*	glycerophosphoryl diester phosphodiesterase Gde1
SPAC13G7.04c	*mac1*	plasma membrane anchored protein, claudin family, predicted membrane sensor Mac1
SPCC306.11	SPCC306.11	Schizosaccharomyces specific protein, uncharacterized
SPNCRNA.1374	*cta3-antisense-1*	antisense RNA (predicted)
SPBC11C11.05	SPBC11C11.05	conserved fungal cell wall protein, Kre9/Knh1 family
SPAC23A1.02c	*ted1*	GPI-remodeling mannose-ethanolamine phosphate phosphodiesterase Ted1
SPAC1006.08	*etd1*	Spg1-binding protein Etd1
SPBC1289.01c	*chr4*	SEL1/TPR repeat protein1, 3-beta-glucan synthase regulatory factor Chf3/Chr4
SPAC1F7.05	*cdc22*	ribonucleoside reductase large subunit Cdc22
SPBC1685.14c	*vid27*	WD repeat protein, Vid27 family, conserved in fungi and plants
SPBC1289.04c	*pob1*	Boi family protein
SPCC576.01c	*xan1*	alpha-ketoglutarate-dependent xanthine dioxygenase Xan1
SPCC1223.09	*uro1*	uricase Uro1
SPAC1002.19	*urg1*	GTP cyclohydrolase II Urg1
SPAC1039.02	SPAC1039.02	extracellular 5′-nucleotidase, human NT5E family
SPCC4B3.10c	*ipk1*	inositol 1,3,4,5,6-pentakisphosphate (IP5) kinase
SPAC56F8.03	*tif52*	translation initiation factor eIF5B Tif52
SPAC29B12.14c	SPAC29B12.14c	plasma membrane purine transmembrane transporter

**Table 11:** Genes upregulated by Atf1 and downregulated by Pcr1.

Gene name	Gene Symbol	Gene Function
SPCC1906.04	*wtf20*	wtf antidote-like meiotic drive suppressor Wtf20
SPAC1834.04	*hht1*	histone H3 h3.1
SPCC1739.15	*wtf21*	wtf meiotic drive antidote-like Wtf21
SPBC1105.12	*hhf3*	histone H4 h4.3
SPAC1834.03c	*hhf1*	histone H4 h4.1
SPAC750.01	SPAC750.01	NADP-dependent aldo/keto reductase, unknown biological role, implicated in cellular detoxification
SPBPB2B2.13	*gal1*	galactokinase Gal1
SPAC1002.19	*urg1*	GTP cyclohydrolase II Urg1

**Table 12:** Genes downregulated by Atf1 and upregulated by Pcr1.

Gene Name	Gene Symbol	Gene Function
SPAPB1E7.04c	SPAPB1E7.04c	chitinase
SPAC19G12.16c	*adg2*	conserved fungal cell surface protein, Kre9/Knh1 family, Adg2
SPCPB1C11.01	*amt1*	plasma membrane ammonium transmembrane transporter Amt1
SPRRNA.02	*rns*	small subunit (15S) rRNA, rns
SPAC821.09	*eng1*	cell septum surface endo-1,3-beta-glucanase Eng1
SPBPB2B2.06c	SPBPB2B2.06c	extracellular 5′-nucleotidase, human NT5E family
SPAC1F8.01	*ght3*	plasma membrane gluconate:proton symporter Ght3
SPBC359.06	*mug14*	adducin, involved in actin cytoskeleton organization
SPAC1F8.03c	*str3*	plasma membrane heme transmembrane transporter Str3
SPAC513.03	*mfm2*	M-factor precursor Mfm2

*Genes upregulated and downregulated in each of the experimental backgrounds are mentioned in the tables below*.

## 2 Materials and Methods

### 2.1 Experimental design

Differential gene expression studies based on RNA sequencing were carried out following overexpression experiments in a series of *S. pombe* transformants and mutants. All samples were processed in duplicates.

### 2.2 Strains, media and growth conditions

*S. pombe* strains used in this study are listed in (Table 13). Cells were grown as described in [8]. For overexpression experiments, cells were grown overnight in Edinburgh Minimal Medium, EMM (Leu-) supplemented with 20 µM thiamine, harvested, washed, resuspended in EMM (Leu-) and incubated for 24 h at 30 °C. Cells were thereafter harvested, washed and resuspended in RNAlater Stabilization Solution (Thermo Scientific).

**Table 13:** List of strains used in the study.

Strain/Plasmid Number	Genotype/Description	Source
GSY001	*h^−^ leu1-32 ura4-D18*	Paul Russel (PR109)
GSY027	*h^−^atf1::ura4^+^*	Kazuhiro Shiozaki (KS1497)
GSY499	*h^+^ leu1 ura4pcr1::ura4^+^atf1::kanMX6*	Elena Hidalgo (MS48)
pGS017	pREP41	Yeast Genetic Resource centre
pGS044	pREP41+Pcr1	Lab Stock [1]

### 2.3 *S. pombe* transformation

1 ml of overnight S. Pombe cultured in YES was harvested and then resuspended in 0.5 ml PEGLET (10 mM Tris [pH 8], 1mM EDTA, 0.1 M lithium acetate, 40% polyethylene glycol [PEG]). 5µl of denatured salmon sperm DNA (10 mg/ml) was added to it. 1 µg of the purified plasmid DNA was then added to this mixture and allowed to stand overnight at room temperature, after which the cells were resuspended in 150 μl YES and spread onto appropriate selection plates.

### 2.4 RNA isolation

TRIzol ™ Reagent (Invitrogen) was used for RNA isolation. After homogenizing the sample with TRIzol ™ reagent, chloroform was added, and the homogenate was allowed to separate into a clear upper aqueous layer (containing RNA), an interphase, and a red lower organic layer (containing the DNA and proteins). RNA was thereafter precipitated from the aqueous layer with isopropanol. Furthermore, the steps of cDNA library preparation and Next Generation Sequencing and Analysis were done by Agrigenome.

### 2.5 Library preparation

TruSeqstranded mRNA sample preparation protocol was used to capture coding RNA and multiple forms of noncoding polyadenylated RNA using poly-T oligo attached magnetic beads. After fragmentation of mRNA, first-strand cDNA was done using reverse transcriptase (strand specificity was obtained by replacing dTTP with dUTP, followed by second-strand cDNA synthesis using DNA Polymerase I and RNase H. Then adenylation of the 3’ ends are done following ligation of adapters. The products are then purified and enriched with PCR to create the final cDNA library. Finally, quality control analysis and quantification of the DNA library templates were performed to create optimum cluster densities across every lane of flow cell.

### 2.6 Data analysis

Raw sequence data generation was done using Fastq [9] file followed by data quality check. Mapping is done to the reference genome using Kim et al [10]. to evaluate sample quality, followed by differential expression analysis using cuffdiff [11,12] Gene Ontology Annotations were assigned using Uniprot [13] and the report of the analysis was produced. Correlation analyses were performed to check the variability between replicates and across samples The box plot was used to show the distribution of data based on the five number summary. Log transformation is performed to make the variation similar across orders of magnitude (See Supplementary Figure S1). The correlation between the samples being compared was revealed by the scatter plot. The samples being compared are said to be highly correlated if the data falls in a straight line (See Supplementary Figure S2). The distance matrix plot showed the correlation between the samples being compared. (See Supplementary Figure S3). The matrix plot describes the number of significant genes at 5% FDR for each pairwise interaction tested. It gives a quick view of the number of significant features at a given q value cutoff <= 0.05 (See Supplementary Figure S4). The Volcano plot helps visualize the statistically significant differentially expressed genes. The plot is constructed by plotting -log10 (*p*-value) on the y-axis, and the log2 fold change between the two samples on the X-axis. Genes that pass the filtering of *q*-value <0.05 are indicated on the plot in red (See Supplementary Figure S5). Further analysis was performed in lab. Genes with significant fold changes were taken for analysis and a cut off of ≥1.5 fold for up-regulated genes and ≤0.75 fold for down-regulated genes was set for further analysis of the differential expression in the gene sets. Gene clusters and functions were generated using DAVID Functional Annotation Bioinformatics tool (David v6.8) [2,3]. Lock et al [14]. was used to assign and verify specific functions of the respective genes. Gene expression profiles during cell cycle and stress were explored using Chen Lab Resources [4,5]. Hulsen et al [7]. application was used for the comparison and visualization of gene lists using area proportional Venn diagrams.

## Funding Information

S.B thanks UGC-URF for fellowship, P.S thanks CSIR for fellowship, S.D. thanks WB-DBT [Ref No. 56(Sanc.)-BT/(Estt.)/RD-17/2017 dated13/08/2018] for fellowship. The authors acknowledge WB-DBT [Ref No. 56(Sanc.)-BT/(Estt.)/RD-17/2017, dated 13/08/2018] for funding.

## CRediT authorship contribution statement

**Sohini Basu:** Methodology, Formal analysis, Data curation, Writing – review & editing. **Priyanka Sarkar:** Formal analysis, Data curation, Writing – review & editing. **Suchismita Datta:** Methodology, Formal analysis, Data curation, Writing – review & editing. **Geetanjali Sundaram:** Methodology, Formal analysis, Data curation, Writing – review & editing.

## Declaration of Competing Interest

The authors declare that they have no known competing financial interests or personal relationships that could have appeared to influence the work reported in this paper.

## Appendix Supplementary materials

Image, application 1

## Footnotes

Supplementary material associated with this article can be found in the online version at doi:10.1016/j.dib.2022.108034.
