# Supplementary material for: Dataset describing the genome wide effects on transcription resulting from alterations in the relative levels of the bZIP transcription factors Atf1 and Pcr1 in Schizosaccharomyces pombe
Source: Data Brief. 2022 Mar 8;42:108034. doi: 10.1016/j.dib.2022.108034 (PMC8960879; doi:10.1016/j.dib.2022.108034)
Supplement: Supplementary file 1 [file mmc1.docx]

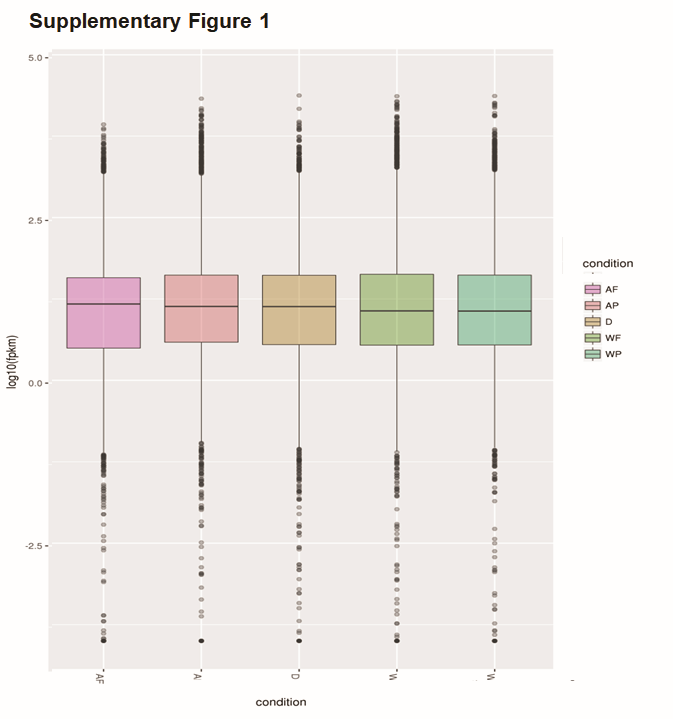


**Figure S1: The box plot represents the distribution of FPKM values**. The x-axis represents samples groups and y-axis represents log10 FPKM value. Log transformation is performed to make the variation similar across orders of magnitude. AF = Δ*atf1*+Ф, AP = Δ*atf1+*Pcr1, D = Δ*atf1*Δ*pcr1,* WF = *wt*+ Ф, WP = *wt*+Pcr1


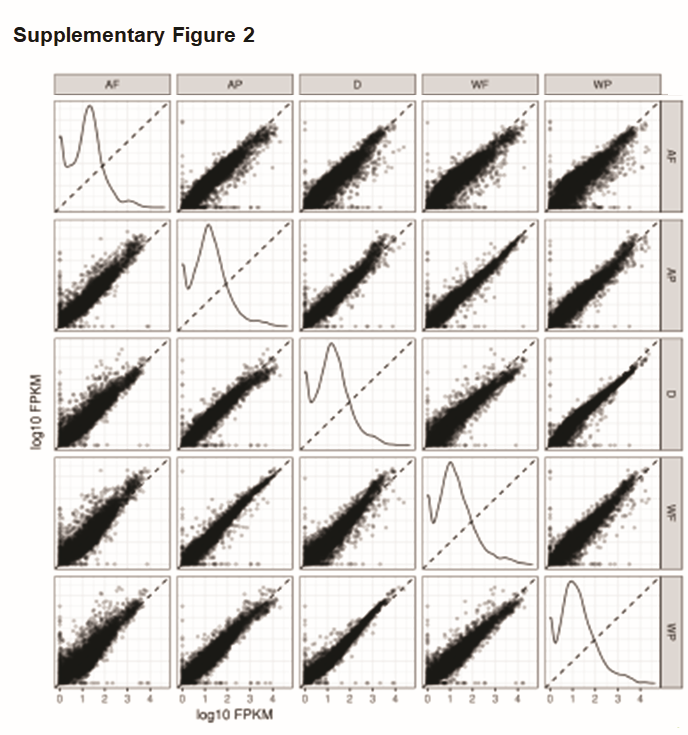


**Figure S2**: **The scatter plot depicted the correlation between the samples being compared**. The x-axis and y-axis represent the log10 transformed FPKM values. The samples were said to have a positive correlation if the data points make a straight line beginning from the origin to high x- and y- values.AF = Δ*atf1*+Ф, AP = Δ*atf1+*Pcr1, D = Δ*atf1*Δ*pcr1,* WF = *wt*+ Ф, WP = *wt*+Pcr1


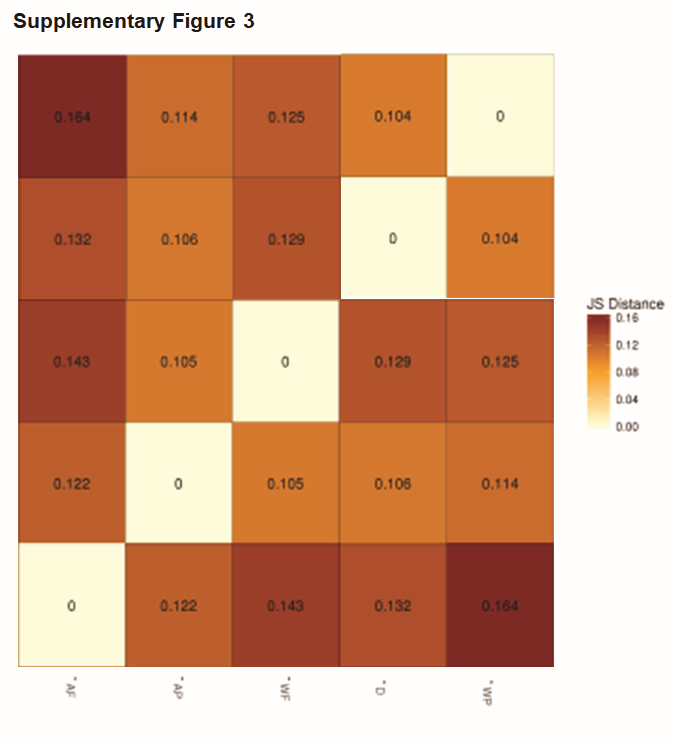


**Figure S3**: **The distance matrix plot showed the pairwise correlation between the samples being compared**. The x-axis and y-axis represent the samples being compared. The samples are highly correlated if the distance is closer to 0.AF = Δ*atf1*+Ф, AP = Δ*atf1+*Pcr1, D = Δ*atf1*Δ*pcr1,* WF = *wt*+ Ф, WP = *wt*+Pcr1


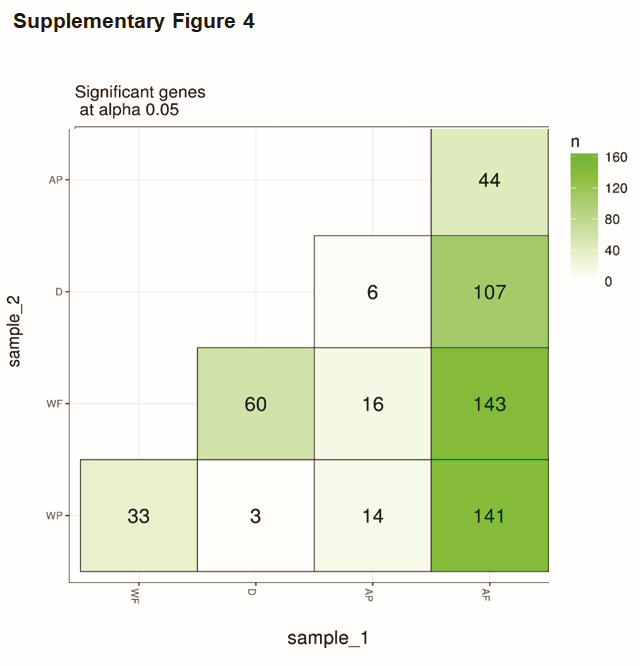


**Figure S4: Representation of the number of significant genes at a 5% FDR for each pairwise interaction tested in a matrix plot**. Significant gene matrix showing the number of significant genes at q-value cutoff <= 0.05.AF = Δ*atf1*+Ф, AP = Δ*atf1+*Pcr1, D = Δ*atf1*Δ*pcr1,* WF = *wt*+ Ф, WP = *wt*+Pcr1


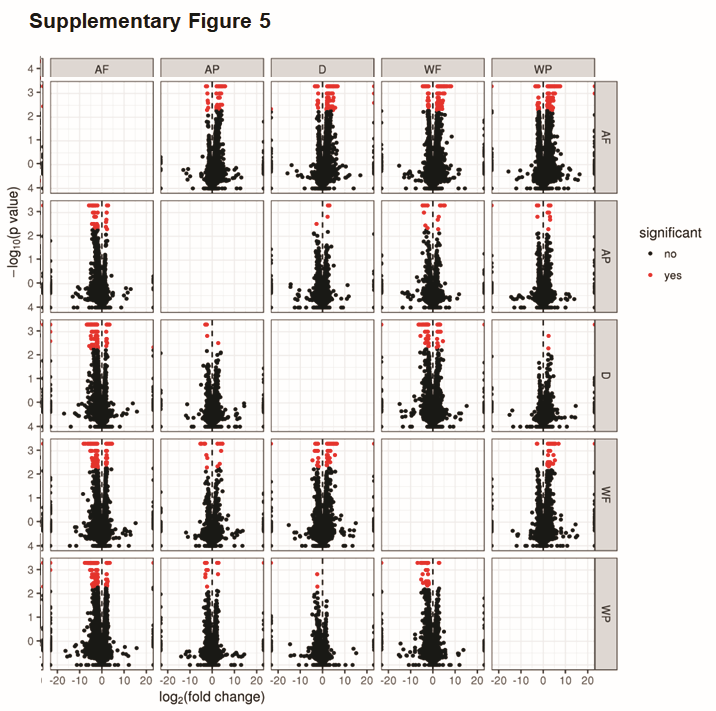


**Figure S5**:**The Volcano plot representing the statistically significant differentially expressed genes**. The plot is constructed by plotting -log10(p-value) on the y-axis, and the log2 fold change between the two samples on the x-axis. This showed the relationship between the p-values and the log2 fold change in normalized expression (FPKM) between the samples. High statistically significant genes are found towards the top of the plot and highly down and up-regulated genes can be found towards the extreme left and right of the plot respectively. The differential gene expression data is used to construct volcano plot.AF = Δ*atf1*+Ф, AP = Δ*atf1+*Pcr1, D = Δ*atf1*Δ*pcr1,* WF = *wt*+ Ф, WP = *wt*+Pcr1
